# Draft Genome Sequences of Marine Bacteria, Degraders of the Sulfated Polysaccharide Ulvan, Extracted from a Green Alga, Ulva meridionalis

**DOI:** 10.1128/mra.01288-22

**Published:** 2023-03-01

**Authors:** Bubai Bhakta, Miho Satoh, Kouhei Ohnishi

**Affiliations:** a The United Graduate School of Agricultural Sciences, Ehime University, Matsuyama, Ehime, Japan; b Research Institute of Molecular Genetics, Kochi University, Nankoku, Kochi, Japan; University of Delaware College of Engineering

## Abstract

We present the draft genome sequences of bacterial strains of the genera *Alteromonas* and *Tenacibaculum*. Polysaccharide utilization loci (PULs) were found in all of the ulvan utilizers. Comparison of the PULs will elucidate the degradation pathway for ulvan.

## ANNOUNCEMENT

The green alga Ulva meridionalis has a high growth rate among *Ulva* species (1), often leading to green tide in coastal sea areas (2). U. meridionalis contains approximately 35% dry weight of ulvan (3), a complex, water-soluble, sulfated polysaccharide that is mainly composed of l-rhamnose-3-sulfate, glucuronic acid, and iduronic acid. Ulvan-utilizing bacteria contain genes involved in ulvan degradation in a gene cluster, called polysaccharide utilization loci (PULs) (4).

U. meridionalis cultivated in onshore aquaculture facilities was hung for 1 week in September 2019 in Uranouchi Bay (Kochi, Japan). The harvested green algae were used to enrich ulvan-utilizing bacteria by incubation at 25°C for 2 days in artificial seawater (Marine Salt Pro; Tetra, Japan) with 0.2% (wt/vol) ulvan extracted from U. meridionalis. Enriched cells were spread on artificial seawater agar with 0.2% (wt/vol) ulvan as the sole carbon source and were incubated at 25°C for 2 days. Isolated bacteria were grown again on the same medium, and we selected seven strains. The V1 to V3 regions of the 16S rRNA gene were amplified from the strains using primers proR2 (5′-AGAGTTTGATCMTGGCTCAG-3′) and 534R (5′-ATTACCGCGGCTGCTGG-3′). The nucleotide sequences of the amplified fragments were determined using a 3130 Genetic Analyzer (Applied Biosystems). The genera identified for the seven strains are listed in Table 1. To identify PULs and to elucidate the evolution of ulvan degradation enzymes, we determined the draft genome sequences of the strains. Chromosomal DNA was extracted from cells that had been grown overnight in half-strength marine broth (Marine Broth 2216; BD Difco) at 25°C using a Wizard genomic DNA purification kit (Promega), fragmented, and prepared with an average size of 330 bp using a QIAseq FX DNA library kit (Qiagen). After adaptors were ligated, the pooled libraries (at a concentration of 20 pM) were sequenced on an Illumina MiSeq system (MiSeq reagent kit v3, 150 cycles), which generated 75-bp paired-end reads. Reads with quality scores of more than 30 were assembled using GS *De Novo* Assembler v3.0. Gene annotation was conducted using the DFAST prokaryotic genome annotation pipeline (5). Default parameters were used for all software unless otherwise specified. Characteristics of the sequenced genomes are listed in Table 1.

**TABLE 1:** Characterization of the genome assemblies of seven ulvan-utilizing bacterial strains

Strain	Genus	GenBank accession no.	DRA accession no.	Genome size (bp)	GC content (%)	Total no. of reads	Coverage (×)	No. of contigs^*a*^	*N*_50_ (bp)	Ulvan lyase genes
KUL106	*Alteromonas*	BLIG00000000	DRA009361	4,587,436	44.5	4,497,972	132	131	115,894	*ullA1*, *ull2*, *ullB1*, *ullB2*, *ullC*
KUL113	*Tenacibaculum*	BLIH00000000	DRA009361	7,597,373	38.6	5,777,600	122	243	72,135	*ullA*, *ullB1*, *ullB2*, *ullB3*, *ullC*
KUL118	*Tenacibaculum*	BLII00000000	DRA009361	7,894,586	39.1	2,189,951	40	289	52,559	*ullA*, *ullB1*, *ullB2*, *ullB3*, *ullC*
KUL150	*Alteromonas*	BLIJ00000000	DRA009361	4,514,422	44.1	1,632,758	47	106	91,645	*ullA*, *ullB1*, *ullB2*, *ullB3*, *ullC*
KUL152	*Tenacibaculum*	BLIK00000000	DRA009361	4,104,732	43.8	1,665,684	60	203	110,934	*ullA1*, *ullA2*, *ullB1*, *ullB2*, *ullB3*, *ullC*
KUL154	*Alteromonas*	BLIL00000000	DRA009361	7,228,898	39.3	5,924,300	63	248	69,681	*ullA1*, *ullA2*, *ullB1*, *ullB2*, *ullB3*, *ullC*
KUL156	*Alteromonas*	BLIM00000000	DRA009361	7,530,308	38.9	8,571,018	42	428	35,018	*ullA1*, *ullA2*, *ullB1*, *ullB2*, *ullB3*, *ullC*

a Longer than 200 bp.

These draft genome sequences of ulvan-utilizing bacteria, along with already published sequences of Nonlabens ulvanivorans (6), *Alteromonas* sp. (7), and *Pseudoalteromonas* sp. (8) strains, will open up the utilization of the polysaccharide ulvan from *Ulva* species. Multiple ulvan lyase genes, including a major long-type PL24 family ulvan lyase (*ullA* [ulvan lyase A]), a short-type PL25 ulvan lyase (*ullB*), and a PL25 ulvan lyase (*ullC*), were found in the genomes of KUL strains (Table 1). No gene for a PL28 ulvan lyase (9) was observed in our strains. We observed several genes related to ulvan degradation surrounding *ullB* and *ullC* in the genomes of KUL strains, and we identified these regions as PULs.

### Data availability.

This whole-genome shotgun (WGS) project has been deposited in DDBJ/EMBL/GenBank. The versions described in this paper are the first versions. The raw reads have been deposited in the DDBJ DRA. The WGS and DRA accession numbers are listed in Table 1.
